# Myelinodegeneration vs. Neurodegeneration in MS Progressive Forms

**DOI:** 10.3390/ijms24021596

**Published:** 2023-01-13

**Authors:** Serge Nataf

**Affiliations:** 1 Bank of Tissues and Cells, Hospices Civils de Lyon, Hôpital Edouard Herriot, Place d’Arsonval, F-69003 Lyon, France; serge.nataf@inserm.fr; 2 Stem-Cell and Brain Research Institute, 18 Avenue de Doyen Lépine, F-69500 Bron, France; 3 Lyon-Est School of Medicine, University Claude Bernard Lyon 1, 43 Bd du 11 Novembre 1918, F-69100 Villeurbanne, France

In MS patients with a progressive form of the disease, the slow deterioration of neurological functions is thought to result from a combination of neuronal cell death, axonal damages and synaptic dysfunctions. Axonal alterations were first reported in the historical neuropathological observations made by Charcot in the 19th century [1]. However, approximately 25 years ago, the notion of inflammation-associated neurodegeneration was put on the front line of MS pathophysiology. Indeed, in the late 1990s, axonal alterations up to the stage of axonal transection were demonstrated in active MS lesions [2,3]. Axonal loss was then found to occur throughout the normal-appearing white matter (NAWM) in patients suffering from a primary progressive or a secondary progressive form of MS [4,5]. Such diffuse axonal alterations were proposed to essentially result from the sum of retrograde and anterograde degenerative processes initiated eitherat sites of axonal transection or from dying neurons [6,7]. Several works also provided evidence that axonal degeneration in MS may stem from a failure of mitochondrial energy metabolism [8,9,10]. On this basis, MS progression was proposed to arise from a diffuse axonopathy targeting predominantly long axons, i.e., axons with a high energy demand [11]. Although diffuse parenchymal inflammation was shown to correlate with diffuse axonal loss [4,5], the neurodegeneration-promoting impact of meningeal inflammation drew increasing interest in the last decade. In particular, neuropathological studies demonstrated that meningeal inflammation correlates with either the extent of neuronal cell loss in MS brains [12,13] or the level of axonal loss in MS spinal cords [14]. Importantly, a causative link between meningeal inflammation and MS-associated neurodegeneration was recently demonstrated in vivo in a rat model of experimentally induced meningeal inflammation [15]. More specifically, the over-expression of lymphotoxin-alpha in meningeal cells was shown to cause the death of cortical neurons and to induce the formation of meningeal tertiary lymphoid structures (TLS) [15]. In this regard, it should be noticed that TLS are essentially observed in MS patients with an accelerated and aggressive course of the disease [16]. Thus, one may argue that mechanisms involved in MS “aggressiveness” may not relate to those involved in MS “progressiveness”. Furthermore, although supported by a substantial amount of data, the neuroinflammation/neurodegeneration hypothesis fails to integrate the existence of diffuse myelin alterations in the central nervous system (CNS) of MS patients. Notably, we and others demonstrated large areas of partial demyelination in periplaque regions, which are associated with low levels of inflammation [17,18,19]. Interestingly, irrespective of localization (brain vs. spinal cord) and plaque activity (chronic–active vs. silent) an important molecular feature shared by periplaques is the down-regulation of *NDRG1*, an oligodendrocyte gene that plays a crucial role in myelin maintenance [20,21]. This observation is reminiscent of previous work that demonstrated the epigenetic silencing of *NDRG1* in “pathology-free” MS brain areas [22]. Additionally, in contrast with the expected inflammatory profile, periplaques exhibit a TGF-beta molecular signature reflecting an anti-inflammatory rather than a pro-inflammatory response [20,21,23]. Other works firmly demonstrated that, beyond periplaques, oligodendrocytes exhibit profound molecular and functional alterations throughout the CNS. This has been demonstrated ex vivo by epigenetics analyses, as mentioned above [22] but also, more recently, via single-cell RNA-seq analyses [24]. Similarly, the in vivo measures of myelin water fraction (MWF) by magnetic resonance imaging (MRI) clearly illustrate the occurrence of diffuse myelin alterations in the brain of MS patients [25,26]. Moreover, in MS patients, such decreased levels of MWF correlate with cognitive decline [27], disability scores [28], and extent of axonal loss [29]. This later point is of particular interest, as it raises the possibility that a process of myelinodegeneration may precede or at least accompany neurodegeneration, as previously proposed [30,31]. Indeed, the functional coupling between oligodendrocytes and axons is now acknowledged, and oligodendrocytes were shown to support axonal energy metabolism via specific glycolytic functions [32,33]. Supporting this view, it was previously shown that mice KO for the myelin genes Mag, Plp or Cnp develop a neurodegenerative process characterized by a diffuse axonopathy [34,35,36,37]. Finally, in mice, genetically determined alterations of myelinating oligodendrocyte are sufficient to provoke or amplify CNS neuroinflammation [38,39].

Future studies should aim to further characterize myelinodegeneration in the CNS of MS patients and determine how diffuse myelin alterations, neuroinflammation and neurodegeneration develop concurrently during the course of MS progressive forms.
